# High-Intensity Interval Training Elicits Higher Enjoyment than Moderate Intensity Continuous Exercise

**DOI:** 10.1371/journal.pone.0166299

**Published:** 2017-01-11

**Authors:** Jacob S. Thum, Gregory Parsons, Taylor Whittle, Todd A. Astorino

**Affiliations:** Department of Kinesiology, California State University San Marcos, San Marcos, California, United States of America; University of Alabama at Birmingham, UNITED STATES

## Abstract

Exercise adherence is affected by factors including perceptions of enjoyment, time availability, and intrinsic motivation. Approximately 50% of individuals withdraw from an exercise program within the first 6 mo of initiation, citing lack of time as a main influence. Time efficient exercise such as high intensity interval training (HIIT) may provide an alternative to moderate intensity continuous exercise (MICT) to elicit substantial health benefits. This study examined differences in enjoyment, affect, and perceived exertion between MICT and HIIT. Twelve recreationally active men and women (age = 29.5 ± 10.7 yr, VO_2_max = 41.4 ± 4.1 mL/kg/min, BMI = 23.1 ± 2.1 kg/m^2^) initially performed a VO_2_max test on a cycle ergometer to determine appropriate workloads for subsequent exercise bouts. Each subject returned for two additional exercise trials, performing either HIIT (eight 1 min bouts of cycling at 85% maximal workload (Wmax) with 1 min of active recovery between bouts) or MICT (20 min of cycling at 45% Wmax) in randomized order. During exercise, rating of perceived exertion (RPE), affect, and blood lactate concentration (BLa) were measured. Additionally, the Physical Activity Enjoyment Scale (PACES) was completed after exercise. Results showed higher enjoyment (p = 0.013) in response to HIIT (103.8 ± 9.4) versus MICT (84.2 ± 19.1). Eleven of 12 participants (92%) preferred HIIT to MICT. However, affect was lower (p<0.05) and HR, RPE, and BLa were higher (p<0.05) in HIIT versus MICT. Although HIIT is more physically demanding than MICT, individuals report greater enjoyment due to its time efficiency and constantly changing stimulus.

***Trial Registration*:** NCT:02981667.

## Introduction

In adults, the recommended amount of physical activity to modify health risks is equivalent to 150 min/wk of moderate intensity continuous exercise (MICT) or 75 min/wk of vigorous exercise [1]. However, in the United States, only approximately 20% of adults meet this recommendation [2]. The greatest barrier to regular physical activity is lack of time ([3–4], which questions the practicality of MICT in the overall adult population considering its relatively large time commitment. High intensity-interval training (HIIT) consisting of repeated, intense exercise bouts separated by passive or active recovery has been widely studied in the last decade ([5]. Data show that compared to MICT, 6 wk of HIIT in the form of repeated Wingate tests elicits similar improvements in maximal oxygen uptake (VO_2_max) and mitochondrial function [6]. In addition, some studies show significantly greater improvements in VO_2_max in response to HIIT compared to MICT [7].

While HIIT may require less time compared to MICT, the shortness of breath, leg pain, and dramatic fatigue usually experienced during HIIT may make this modality less tolerable in certain populations. The Dual-Mode theory [8] demonstrates that at intensities below the ventilatory threshold (VT), affect as measured with the Feeling Scale [9] is positive, suggesting that exercise at this intensity is pleasurable and long-term may promote exercise adherence [10]. However, at supra-VT work rates which are characteristic of HIIT, affect becomes less positive due to onset of interoceptive cues and greater contribution of anaerobic metabolism to ATP supply [8]. This may ultimately reduce exercisers’ willingness to partake in more intense exercise, which is important for designing proper exercise prescription especially when more intense exercise may elicit superior improvements in VO_2_max [11].

A few studies have compared perceptions of affect and/or enjoyment in response to acute bouts of HIIT versus MICT. Bartlett et al. [12] reported that running-based HIIT led to greater perceived enjoyment compared to MICT in active men. When average intensity was matched between HIIT and MICT (intensity equal to 85% respiratory compensation point), young healthy men reported higher RPE and fatigue, lower affect, but similar enjoyment in response to HIIT versus MICT [13]. In untrained adults, HIIT was reported to be less pleasurable than MICT [14], but more than 50% of participants reported that they preferred it. In addition, HIIT was viewed as more enjoyable than vigorous exercise yet no differences in enjoyment were seen compared to MICT. In obese young women, Kong et al. [15] reported that enjoyment was significantly higher during each week of a 5 wk HIIT regimen (repeated 8 s “all-out” sprints separated by 12 s passive recovery) compared to MICT (40 min at 60–80%VO_2_max).

Despite empirical evidence showing significant adaptations and potentially higher enjoyment in response to HIIT compared to MICT, acceptance of high intensity interval training is not universal. Recently, Hardcastle et al. [16] criticized the practicality of one paradigm of HIIT, sprint interval training (SIT) characterized by repeated Wingate tests, which has been used to improve VO_2_max and other health outcomes [6,17]. Hardcastle et al. [16] assert that SIT is too physically demanding for a largely sedentary population, and that low levels of enjoyment and affect elicited by the intense nature of SIT may discourage exercise adherence and motivation. However, the current study and related investigations ([12–14] incorporated HIIT which has a lower metabolic perturbation than SIT. Despite significant physiological differences between these modes of interval training, findings from recent studies showed similar enjoyment between two regimes of HIIT [18] and similar affect between HIIT and SIT [19].

Due to these equivocal results, it remains to be determined whether HIIT is a more enjoyable and preferable modality of exercise compared to MICT. This line of inquiry is necessary to identify effective, time-efficient, and enjoyable modes of exercise to impact fitness and overall health status in various populations.

The aim of this study was to examine differences in enjoyment, perceived exertion, and affect between MICT and HIT in recreationally active individuals. It was hypothesized that HIIT will be perceived as more enjoyable compared to MICT, although lower affect and higher RPE were anticipated in response to HIIT.

## Methods

### Ethics Statement

Prior to providing written informed consent, all participants filled out a health-history questionnaire to ensure that they met all inclusion criteria, and all procedures were approved by the CSU—San Marcos Institutional Review Board (S1 protocol).

### Design

This repeated measures, within-subjects study required all participants to complete three sessions of exercise in a climate-controlled laboratory (temperature and relative humidity equal to 19–22°C and 40–60%, respectively). Initially, VO_2_max was assessed on a cycle ergometer during progressive exercise to exhaustion. Between 48–96 h later at the same time of day, participants initiated one of two bouts of either HIIT or MICT during which perceptual responses and blood lactate concentration were obtained. Participants refrained from intense exercise for 48 h before each session, and they completed a dietary log so food intake was standardized 24 h before each session. In addition, all subjects fasted for 3 h prior to each session. The STROBE checklist is available to the reader as S1 checklist.

### Participants

**Fig 1:**
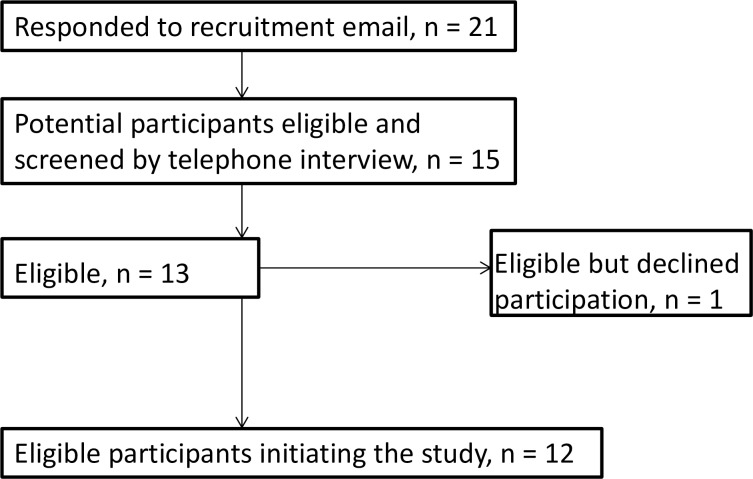
Flow chart describing participant recruitment.

Healthy, non-obese (BMI < 30 kg/m^2^), recreationally active men (n = 8) and women (n = 4) were recruited via word-of-mouth from the university population from September to December 2015. Their characteristics are listed in Table 1. Participants performed a minimum of 3 h/wk of physical activity including aerobic exercise, resistance training, surfing, group exercise, hiking, and/or non-competitive sport and had done so for at least 1 yr. None reported any fitness-related goals such as training for a particular event. Only one participant regularly performed cycling-based HIIT similar to that performed in the current study. Additional inclusion criteria were non-smokers between the ages of 18–49 yr who lacked knee ailments or any other pre-existing health conditions. A recruitment flowchart for the study is shown in Fig 1.

**Table 1:** Physical characteristics of participants (N = 12).

Variables	Mean (SD)	Range
Age (yr)	25.5 (10.7)	18–49
Gender	NA	8 men/4 women
Height (cm)	173.3 (8.9)	162–191
Body mass (kg)	69.9 (14.5)	55.5–100.0
Body mass index (kg/m^2^)	23.1 (3.0)	20.9–28.6
Percent body fat (%)	19.6 (2.5)	15.1–25.2
VO_2_max (mL/kg/min)	41.3 (4.9)	35.0–47.6
Maximal heart rate (b/min)	179.5 (13.9)	161–200
Maximal workload (W)	271.8 (42.4)	221–352

### Baseline Assessment

On the first visit, participants underwent anthropometric measurements including height, weight, and body composition. Skinfold (Lange, Santa Cruz, CA) measurements were performed twice in rotational order at each of three sites (chest, abdomen, and thigh for men; triceps, suprailiac, and thigh for women), and the mean value was used to estimate percent body fat using the appropriate equation [20–21]. Next, the participant initiated incremental exercise on an electrically-braked cycle ergometer (Velotron DynaFit Pro, RacerMate, Seattle, WA) to determine VO_2_max using a ramp protocol. Work rate began at 50–70 W for 2 min followed by 25–35 W/min increases in work rate until volitional fatigue. During exercise, heart rate (HR) was assessed via telemetry (Polar, Woodbury, NY) and gas exchange data were obtained every 15 s using a metabolic cart (ParvoMedics True One, Sandy, Utah) which was calibrated pre-exercise according to manufacturer guidelines. Maximal workload (in Watts) from this bout was used to determine the exercise intensities of subsequent trials. VO_2_max incidence was confirmed using established criteria [22] including respiratory exchange ratio > 1.15, leveling off in VO_2_ at exercise termination, and HR within 10 b/min of the age-predicted value (220 –age).

### Completion of HIIT and MICT

At the same time of day as the VO_2_max trial within subjects, participants performed either HIIT or MICT, whose order was randomized using a Latin Squares design [23]. Trials were separated by a minimum of 2 d to maximum of 7 d. Exercise began with a 5 min warmup at 25%Wmax. High intensity interval training consisted of eight 60 s bouts of cycling at 85%Wmax separated by 60 s of recovery at 25%Wmax. During MICT, participants pedaled at 45%Wmax for 20 min. Across regimens, exercise duration was slightly different (16 vs. 20 min).

### Assessment of Perceptual Responses

Rating of perceived exertion (RPE, Category Ratio-10, 11-point scale))[24], and affect (11-point scale, rating from +5 very good to -5 very bad) [9] were recorded pre-exercise while participants were seated on the ergometer and at 25, 50, 75, and 100% of session completion. During HIIT, these were measured at cessation of each 60 s interval in the order of RPE followed by affect. Before each trial, participants were read specific instructions according to what each measure encompassed. The meaning of the CR-10 scale was communicated by instructing participants to report perceptions of their exertion in terms of their breathing, heart rate, and level of fatigue. For affect, they were read the following text: *While participating in exercise*, *it is common to experience changes in mood*. *Some individuals find exercise pleasurable*, *whereas others find it to be unpleasant*. *Additionally*, *feeling may fluctuate across time*. *That is*, *one might feel good and bad a number of times during exercise*. They were asked to respond to each scale in terms of how they felt at that moment.

After a 5 min cool-down, subjects were seated for 5 min and subsequently completed the Physical Activity Enjoyment Scale (PACES) [25]. The 10 min period between the cessation of exercise and completion of PACES was necessary to ensure focus by mitigating effects of residual fatigue on participants’ score. This scale was used to assess level of enjoyment of HIIT and MICT using participant responses to 18 questions scored on a 1–7 Likert scale. After completion of both trials, participants were asked which modality of exercise they ultimately preferred (HIIT or MICT).

### Assessment of Blood Lactate Concentration and Heart Rate

Blood lactate concentration (BLa) was recorded during MICT and HIIT at the following intervals: before the warm-up, at 25% and 75% of exercise completion, and 3 min post-exercise. During HIIT, blood was sampled immediately after the completion of training bouts 2 and 6. Blood samples were acquired with a portable analyzer (Lactate Plus, Nova Biomedical, Waltham, Massachusetts) and fingerstick lancets (Unistix 3 Comfort, Owen Mumford, Marietta, GA). Heart rate was measured continuously during exercise using telemetry.

### Statistical Analysis

Data were expressed as mean ± standard deviation (SD) and were analyzed using SPSS version 20.0 (Chicago, Illinois). A two-way (time and exercise regime) analysis of variance (ANOVA) with repeated measures was used to compare differences in outcome measures between MICT and HIT. If a significant F ratio was obtained, Tukey’s post hoc test was used to identify significant differences between means. A paired t-test was used to determine significant differences in enjoyment between exercise modalities. At a power equal to 0.80 (two-tailed α = 0.05) and effect size for enjoyment equal to 1.0, a minimum sample size of eight individuals was determined [26] which is similar to a previous study [12]. Effect size was reported as Cohen’s d. Significance was set at p<0.05.

## Results

**Fig 2:**
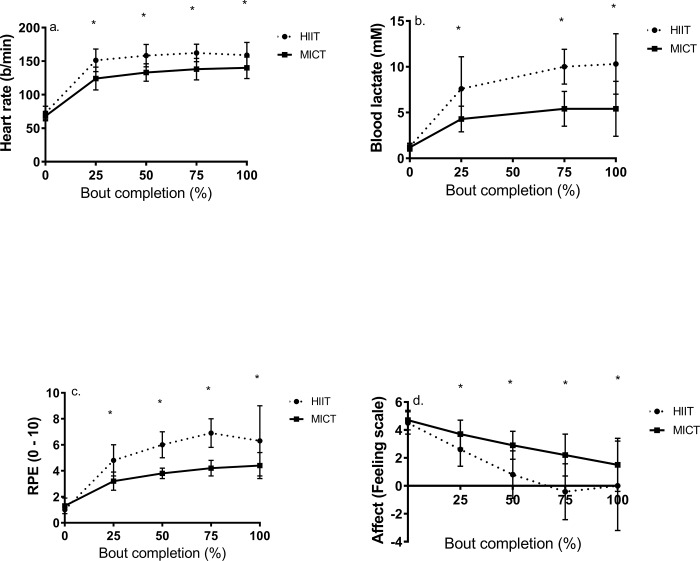
Change in a) heart rate, b) blood lactate concentration, c), rating of perceived exertion and d) affect (mean ± SD) in response to high intensity interval training versus continuous exercise. * = p<0.05 versus MICT.

All participants completed all bouts without incident. To confirm the higher absolute intensity of HIIT versus MICT, Fig 2A and 2B reveal change in HR and BLa during exercise. For HR, there was a regimeXtime interaction (F_4,40_ = 10.0, p<0.001, d = 4.08). Post hoc analyses showed significantly higher HR at all time points in HIIT versus MICT. Peak intensity of MICT (77%HRmax) was lower than that of HIIT (89%HRmax). Similar findings were shown for BLa, indicating a significant regimeXtime interaction (F_3,30_ = 14.6, p<0.001, d = 1.0). Post hoc analyses showed that all exercise values of BLa were higher during HIIT versus MICT.

### Differences in Physical Activity Enjoyment in Response to HIIT versus MICT

PACES scores indicated a significant difference in enjoyment (p = 0.013, d = 1.54) between MICT (84.2 ± 19.1) and HIIT (103.8 ± 9.4). Ninety two percent of participants demonstrated higher enjoyment and preference for HIIT compared to MICT.

### Differences in RPE and Affect in Response to HIIT versus MICT

A significant regimeXtime interaction (F_4,40_ = 9.6, p = 0.003, d = 0.74) was demonstrated for RPE. Post hoc results showed that all exercise RPE values were higher by 1.5–2.7 units in HIIT compared to MICT (Fig 2C). Affect declined during exercise (F_4,40_ = 19.6, p<0.001) to values at exercise cessation equal to 1.5 ± 1.9 and 0.0 ± 3.2 in MICT and HIIT, respectively. Data showed a significant regimeXtime interaction (F_4,40_ = 7.1, p = 0.004, d = 0.78). Post hoc analyses showed that all exercise affect values were less positive in response to HIIT versus MICT (Fig 2D).

## Discussion

Given the rising epidemic of sedentarism in American society [27], it is increasingly important for exercise professionals to identify various modalities of exercise that are enjoyable, time efficient, and produce significant health benefits. According to Burgomaster et al. [6], interval training has been shown to elicit comparable physiological adaptations to endurance training, despite a much lower training volume and time commitment. Although a lower time commitment of exercise may have the potential to increase exercise adherence, the extent to which individuals enjoy HIIT remains unclear. This study examined differences in enjoyment in response to cycling-based HIIT compared to MICT. Our data showed greater enjoyment of HIIT compared to MICT despite higher metabolic stress in the form of significantly higher RPE, HR, and BLa.

Unlike previous studies in this area [13–14,28], we examined differences in BLa between MICT and HIIT. Our data show significant increases in BLa for both exercise regimes, although values were significantly higher in HIIT compared to MICT (Fig 1B). This partially supports the less positive affect exhibited throughout and at cessation of HIIT versus MICT, which is explained by the Dual-Mode Theory [8]. It is apparent [8] that affect begins to decline with increasing reliance on anaerobic metabolism which would be more substantial at intensities equal to 85%Wmax versus 45%Wmax as performed in MICT.

Yet despite this more aversive response consequent with HIIT, enjoyment as measured with the PACES scale was significantly higher compared to higher-volume MICT. Three potential factors explain this response. First, the intermittent nature of HIIT provides participants with multiple recovery intervals which may provide a “break” from less positive affective responses [14] which are seen with continuous exercise at supra-VT intensities. Second, many participants viewed MICT as quite monotonous and did not enjoy maintaining a moderate effort for 20 min. In contrast, HIIT provides an ever changing stimulus that breaks the entire session up into small increments of work interspersed with recovery. Third, there is a certain level of accomplishment that is experienced during HIIT which is not apparent in MICT [14]. Overall, this may give the exerciser a greater amount of self-confidence and may explain why 92% of participants preferred HIIT to MICT.

Various methodological differences inherent in previous studies need to be considered when trying to compare findings across studies. In the Bartlett et al. study [12], participants were highly-fit men with a VO_2_max equal to 57 mL/kg/min who completed a 50 min session of MICT at 70%VO_2_max or HIIT consisting of six 3 min intervals at 90%VO_2_max. Work and average intensity were matched across bouts. Enjoyment as measured with PACES was higher by approximately 25 units in response to HIIT compared to MICT. However, these findings are not unexpected considering that this sample of participants likely has a history of positive responses to intense exercise participation. In a subsequent study in 15 young men (VO_2_max = 47.9 mL/kg/min) [13], repeated 2 min intervals at 100%VO_2_max led to less positive affect and greater fatigue versus MICT consisting of 24 min of cycling at 72%VO_2_max, although enjoyment was similar. In the Jung et al. study [14], 44 inactive men and women (VO_2_max = 36 mL/kg/min) performed in randomized order HIIT consisting of 1 min bouts at 100%Wpeak, and moderate (40 min at 40%Wpeak) and vigorous MICT (20 min at 80%Wpeak). Despite a less positive affect reported in HIIT versus moderate MICT, participants exhibited similar task self-efficacy and greater enjoyment versus vigorous exercise (p = 0.01) and MICT (p = 0.08). An examination of these data and ours (Fig 1D) suggests some interesting trends. Lower intensity HIIT (~78%Wpeak) as performed in a previous study [28] elicits more positive affect values during exercise equal to 2.0 (between fairly good and good) compared to HIIT used in the present study (85%Wpeak) and that of Jung et al. (100%Wpeak) [14], in which affect reached a nadir to values near 0 (neutral). When HIIT bouts are sustained for up to 2 min at 100%VO_2_max [13], affect declined to values of approximately -2.5 (bad). Although speculative, this suggests an inverse association between the specific work rate employed in HIIT and the affectual response to exercise.

A recent study [19] showed similar affect in response to a single bout of sprint interval training (SIT) with a 1:3 ratio of work to recovery compared to HIIT with a 1:1 ratio, although BLa and RPE were higher in response to SIT. Nevertheless, enjoyment was not assessed in this study. Similarly, there was no difference in enjoyment between dissimilar HIIT regimes (16 X 1 min at 90–95%HRpeak versus 4 X 4 min at 90–95%HRpeak) in young men [18]. As intensities of HIIT can widely vary from as low as 60%Wmax [29] to 300%Wmax [6], there is a potential for differences in physiological and perceptual responses across this continuum. Future studies should explore the optimum intensity and/or work:recovery interval enhancing levels of enjoyment and affect in HIIT compared to MICT, considering that in-task affect is related to intentions to exercise [30]. In addition, a few studies have examined changes in enjoyment during chronic administration of HIIT. In young, healthy obese men and women [31], enjoyment significantly increased during training, and in another study [15], enjoyment was consistently higher throughout 5 wk of HIIT versus MICT despite it being at a higher intensity (80%VO_2_peak vs. 71%VO_2_peak). These findings, albeit preliminary, show that chronic HIIT is viewed as more enjoyable than MICT and emphasize the potential of this exercise paradigm in young, overweight-to-obese individuals.

The structure of HIIT in the present study followed a 1:1 ratio of work to recovery. This ratio was also used in sedentary adults [14,29] and in overweight to obese inactive adults [28]. In contrast, a 2:1 ratio was used in the study by Oliveira et al. [13] in which many participants could not complete the interval protocol, which may have led to greater fatigue compared to MICT. Martinez et al. [28] revealed that interval exercise can be pleasurable even in sedentary individuals. In this study, 20 insufficiently active (BMI = 25–35 kg/m^2^) individuals completed a trial of heavy continuous exercise (20 min at 50%Wmax) and three bouts of HIIT: 30s work/30s rest, 60s work/60s rest, and 120s work/120s rest. Interval sessions were 24 min in duration and were completed at an intensity equal to 60% of the difference between anaerobic threshold and VO_2_max, which was equivalent to 78%Wmax. Affect and enjoyment were assessed during and after exercise. Greater enjoyment and affect (p<0.05) were reported for 30 and 60 s HIIT bouts compared to the 120 s duration and continuous exercise, which were seen as significantly more aversive. These findings suggest that interval duration ≤ 60 s and having a 1:1 ratio of work:recovery may be optimal for increasing perceptual responses to exercise and potentially long-term adherence to this modality of training.

Limitations of the current study included a relatively small sample consisting of habitually active men and women varying in age and gender. Despite this small and heterogeneous sample, differences in outcome measures were highly significant between regimes. In addition, the inclusion of both genders, which was not done in previous studies [12–13], broadens our findings to a more diverse population. Similar to our results, Jung et al. [14] and Martinez et al. [28] also showed significantly higher enjoyment in response to HIIT compared to MICT in a large sample of men and women, which would suggest that men and women consistently report higher enjoyment of HIIT. However, their participants were inactive and those in the current study were habitual exercisers, which suggests that fitness level may not significantly alter enjoyment responses to low-volume HIIT. Nevertheless, individual differences in affective responses exist [14], so further study is merited to examine if differences in gender and fitness level mediate the enjoyment response to various intensities of exercise including HIIT. It remains to be determined to what extent perceptual responses such as higher enjoyment and lower affect in HIIT are influenced by the timing of inquiry. It is possible that a greater sense of accomplishment is realized after rather than during HIIT, altering perceptions of enjoyment and mood. The entire HIIT session performed in the present study was 4 min shorter in duration than MICT, making it more time efficient. In addition, the unequal disparity of male and female participants does not allow us to consider a potential role of gender on these responses, as done in a previous study [32]. Although enjoyment responses to a single bout of HIIT may be predictive of attitudes towards chronic exercise participation in this modality, little research [15, 31] exists examining if enjoyment is modified long-term. Lastly, sprint interval training [33–34] is a widely used and more intense approach to HIIT whose completion improves various health outcomes; however, it was not completed in the present study. Consequently, additional research is merited to examine if this modality elicits different perceptions of enjoyment compared to MICT or HIIT.

## Conclusion

Data reveal that HIIT requiring a shorter time commitment than MICT led to greater enjoyment in a small sample of active men and women. Despite greater metabolic stress, over 90% of participants preferred HIIT versus MICT. These findings may support increasing integration of HIIT into the regular exercise routine of healthy, active individuals. Further study in larger heterogeneous populations should attempt to substantiate our findings and investigate if the greater enjoyment in response to low-volume HIIT compared to MICT is also revealed at all intensities above VT as well as when work:recovery interval is manipulated.

## Supporting Information

S1 Checklist STROBE checklist. (DOCX)

S1 Protocol IRB protocol. (PDF)
